# Developing physical activity counselling in primary care through participatory action approach

**DOI:** 10.1186/s12875-016-0540-x

**Published:** 2016-10-04

**Authors:** Minna Aittasalo, Katriina Kukkonen-Harjula, Erja Toropainen, Marjo Rinne, Kari Tokola, Tommi Vasankari

**Affiliations:** 1 UKK Institute for Health Promotion Research, P.O. Box 30, Tampere, FI-33501 Finland; 2 South Karelia Social and Health Care District (Eksote), Valto Käkelän katu 3 B, Lappeenranta, FI-53130 Finland; 3 University of Tampere, School of Health Sciences, Tampere, FI-33014 Finland; 4 National Institute for Health and Welfare, P.O. Box 30, Helsinki, FI-00271 Finland

**Keywords:** Physical activity, Counselling, Implementation, Primary care, Developing

## Abstract

**Background:**

Many adults are insufficiently physically active for health. Counselling is the main method to promote physical activity (PA) in primary care but often implemented inadequately. The aim of this study was to increase health professionals’ i) know-how about health-related PA and PA counselling, ii) implementation and quality of PA counselling, iii) familiarity with and use of Physical Activity Prescription (PAP), iv) internal and external collaboration and v) use of electronic patient record system in PA counselling.

**Methods:**

Four Finnish health centres participated. Each nominated a working group for reaching the goals through a 6-month development work, which was supported with monthly tutorials by the research group. The outcome evaluation of the development work included 19 variables, which reflected the five goals and were assessed before (baseline) and after the development work (follow-up). Variable-specific differences in proportions (%) and their 95 % confidence intervals (CI) between the time points indicated change. The measures were questionnaires to the health professionals (*N* = 75 at baseline and *N* = 80 at follow-up) and patients (*N* = 441 and *N* = 431), professionals’ record sheets on patient visits (N = 1008 and *N* = 1000), and telephone interviews to external partners (*N* = 48 and *N* = 28). The process was evaluated with the extent the working group members participated in the development work and with the implementation of development actions. Assessment was based on meeting minutes of tutorials and working group meetings.

**Results:**

Health professionals’ familiarity with PAP (questionnaire, change 39 %-points; 95 % CI 26.5 to 52.5) and use of PAP (questionnaire, 32 %-points; 95 % CI 18.9 to 45.1 and record sheet, 4 %-points; 95 % CI 2.7 to 5.3) increased. A greater proportion of professionals had agreed in their working unit on using PAP (questionnaire, 32 %-points; 95 % CI 20.3 to 43.7) and used PAP as a referral to other health professionals (record sheet, 1 %-point; 95 % CI 0.3 to 1.7). Also the know-how of PA and PA counselling showed improvement but not statistically significantly. The working group members participated unevenly in the development work and had difficulties in allocating time for the work. This was seen in limited number of actions implemented.

**Conclusions:**

The study was able to achieve some improvements in the familiarity with and use of PAP and to lesser extent in the know-how of health-related PA and PA counselling. To observe changes in other goals, which targeted more at organisational, inter-professional and multi-sectorial level, may have required more long-term actions.

**Electronic supplementary material:**

The online version of this article (doi:10.1186/s12875-016-0540-x) contains supplementary material, which is available to authorized users.

## Background

Globally approximately 30 % of adults are insufficiently physically active for their health [1]. Physical inactivity increases the risk of e.g. atherosclerotic diseases, type 2 diabetes, and breast and colon cancer [2]. New findings suggest that sedentary behaviour, e.g. sitting continuously for hours, is an independent health risk regardless of whether the person meets physical activity (PA) recommendations or not [3]. According to recent multi-country surveys, adults sit on average 5 to 6 h per day [4] and approximately 20 % of the population spend more than seven and a half hours of the day in sitting [5].

Health care is one of the most important settings for promoting PA [6]. Counselling, in turn, is the most acceptable and familiar way for health professionals to bring up lifestyle issues at the appointments. The aim of PA counselling is to develop the patient’s own views and skills to support his or her health, wellbeing and functional capacity [7].

In general, health professionals have a positive view regarding the need of PA counselling [8]. Nevertheless, PA is promoted in Finland [9] and internationally [10, 11] in only a fraction of patient visits where it would be justified. Obstacles among health professionals include e.g. lack of time, personal know-how and patients’ motivation [8, 12]. In some studies health professionals’ age, sex, profession, practice experience, physical health, PA level and education on PA promotion have also been found to be associated with the provision of PA promotion but the relationships seem yet inconclusive [12].

No single organization, professional group or professional has the opportunity to take over the entire, often quite lengthy PA counselling process. The responsibility can be shared amongst the primary healthcare team. Inter-sectorial collaboration between primary care, municipal PA services, sports clubs, fitness centres and community centres is also important [13, 14].

**Fig. 1:**
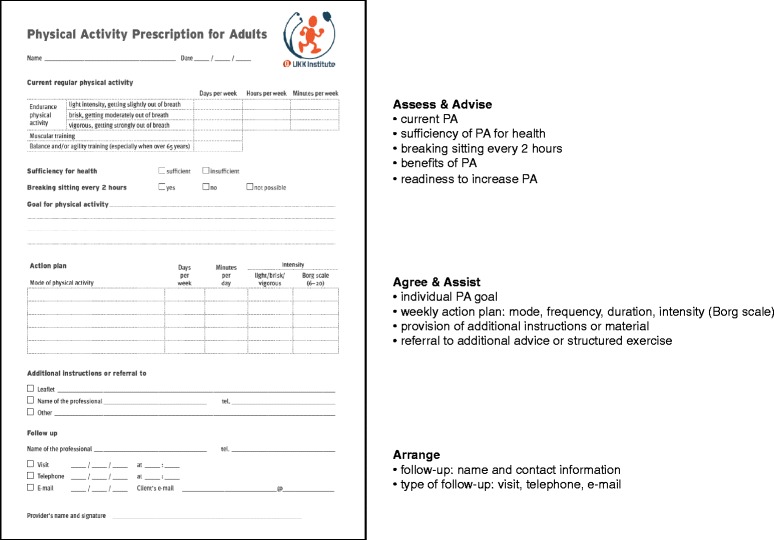
Physical Activity Prescription (PAP) within the framework of 5A’s (Estabrooks et al. [16])

In order to develop local practices and service paths of PA counselling in Finland, a national Physical Activity Prescription Program (PAPP) was implemented in 2001–2004 [15]. In short, the program was implemented in collaboration with two research organisations, two patient associations, Finnish Medical Association and a government funded PA program. PAPP included material and tool development, training of physicians and different ways of popularising PA counselling to health and exercise professionals and stakeholders. As part of material and tool development the Physical Activity Prescription (PAP) (http://www.ukkinstituutti.fi/en/products) was developed to provide especially physicians with a best practice protocol for PA counselling in the primary care setting. The tool was based on the principles of 5A’s [16], which has been recommended as a clinical practice guideline for brief behaviour change counselling by e.g. the Task Forces of Preventive Health Services in Canada [17] and US [18] (Fig. 1). In a randomized trial conducted after the program in 24 primary care units in Finland PAP was shown effective in increasing moderate-intensity PA of insufficiently physically active patients by approximately one weekly session at two and six months’ time interval [19]. The results were consistent with international studies on ‘brief counselling’ on PA [20].

However, based on RE-AIM evaluation [21] the national PAPP did not manage to influence local PA counselling practices [15]. To increase the use of PA and other written material in PA counselling the duration of the program should have been longer and more effort should have been invested in: (i) strengthening physicians’ confidence in PA counselling and knowledge about the effectiveness of PA counselling at the national level and (ii) facilitating inter-sectorial cooperation in adopting PAP as a counselling tool at the local and regional level.

The findings are in line with implementation theories [22] and research [23], which indicate that the adoption of new methods is a complex phenomenon with multiple attributes and that implementation should be tailored to the characteristics of an organisation and staff. As a result, local actions and partnerships are likely to be more effective in order to transfer evidence-based practices to clinical work [24, 25].

### Aim of the study

In the light of findings from PAPP and studies on implementation the aim of the current study was to help health centres to develop their PA counselling practices by offering them local support for the implementation. For this purpose each participating health centre nominated a working group to plan and carry out development actions, which would best suit for their own contexts. The working groups were supported with monthly tutorial meetings held by one of the researchers (ET). Thus, the development work included tutorials, working group meetings between the tutorials and all the actions that the working groups implemented during the study to develop PA counselling.

The specific goals of the development work in each health centre was to increase the primary care professionals’

i) know-how of health-related PA and PA counselling
ii) implementation and quality of PA counselling
iii) familiarity with and use of PAP
iv) internal and external collaboration in PA counselling and
v) use of electronic patient record system in PA counselling.

## Methods

The study plan was approved by the Ethics Committee of the Tampere Region, under the auspices of University of Tampere, Human Sciences (http://www.uta.fi/english/research/ethics/review/committee.html, running number 1/2010).

### Health centres and study procedure

**Fig. 2:**
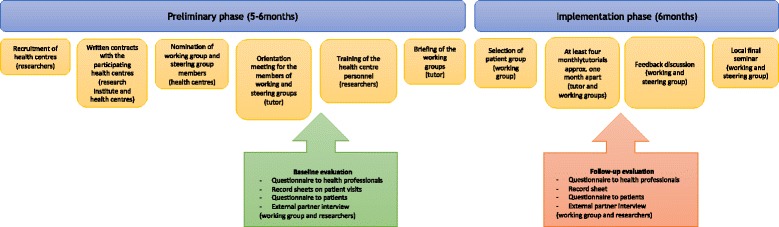
Study phases and procedure. Responsible parties shown in brackets

The study was divided into preliminary and implementation phase (Fig. 2). *In the preliminary phase* (5 to 6 months), voluntary health centres were recruited with an information leaflet sent via email to contact persons responsible for coordinating type 2 diabetes care in the 23 municipalities of the Pirkanmaa region, Finland. After that a more detailed introduction was provided to the four health centres expressing interest, and a written contract on participation was made with all of them. In Finland, health care is provided on the basis of residence and financed with general tax revenues. Primary health care services are the responsibility of municipalities and are offered through local health centres. Each municipality has a health centre but some small municipalities may share resources with a neighbouring municipality. Basic services are defined by law. Private-sector complements the public services and reimbursement from using private services can be applied under the Health Insurance Act.

Each participating health centre appointed a multi-professional working group to plan and implement development work and a local steering group to give support to the working group and disseminate the yields of the working group to PA counselling actors outside health care. For this reason, the health centres were encouraged to recruit and engage representatives from municipal PA services, sports clubs, community centres and private fitness centres to the steering group. The working group and steering group were orientated to the study in a joint meeting. After the orientation, the researchers (ET, TV/KK-H, MA) held a two-hour training session with the health centres’ employees about the study, the activities of the working group, health-related PA, PA counselling (principles of counselling including 5A’s, health behaviour change) and PAP (introduction to the material, practical training in pairs of how to use PAP). The most important target groups of the training were physicians, nurses, public health nurses and physiotherapists because they are the most important providers of PA counselling. In this report, collective term ‘nurse’ is used for nurses and public health nurses. After the training, the tutor, which was one of the members of the research group (ET), briefed the working group on the procedure and assessment of the development work. The working groups nominated a person amongst them to handle contact with the tutor and health centre management.

*The implementation phase* (6 months) was based on the principles of participatory action research [26] and co-operative planning [27]. Each working group selected one adult patient group (e.g. with hypertension, type 2 diabetes, obesity, high cholesterol level, low back pain etc.) as a target group for the development work. At this stage only one patient group was chosen to avoid excessive workload to the members of the working group. The intention was that after the study the health centre would start developing PA counselling in the other patient groups as well. The health centres varied in size, financial and staff resources, organisational factors and type of population that they served. Therefore, each working group was free to choose any patient group, which was considered important and within the limits of staff resources.

The working group received support for their development work through at least four tutorial meetings (Table 1). Two hours were reserved for each tutorial meeting, and their general content was outlined in advance. The tutor took observation notes and the working groups wrote meeting minutes of the tutorials. The working groups were encouraged to utilize the meeting minutes in informing other health centre staff about the proceedings of the development work.

**Table 1:** Goals and contents of the working group meetings

Meeting	Goal	Contents
Orientation^a^	Preconditioning	- Agreeing on the roles and responsibilities of the working group - Outlining the principles of the development work and tutorial meetings - Overviewing the current practices on physical activity counselling based on the preliminary results from the baseline questionnaire to the professionals
1st tutorial	Planning	- Determining the patient group - Choosing goals and actions
2nd tutorial	Implementation	- Reviewing goals and actions - Agreeing on practical arrangements related to the implementation
3rd tutorial	Revision Collaboration	- Revising goals and actions - Starting inter-sectorial collaboration with external partners
4th tutorial	Evaluation Communication	- Evaluating the outcomes of the development work - Planning the protocol for the inter-sectorial collaboration - Planning of the feedback discussion
Feedback discussion^a^	Maintenance Dissemination	- Summarizing the development process - Presenting the outcomes of the development work - Application of the results of the development work to another patient group

^a^Also attended by the members of the steering group

Between the tutorial meetings, the members of the working groups were asked to allocate approximately 2 to 4 h per week for reviewing and carrying out the actions related to the development work. Each working group selected the actions on the basis of tutorial discussions. Thus, the actions were tailored according to the needs and resources of each individual health centre. The actions were supported with written material developed by the research institute, such as ‘physical activity pie’ posters, PA counselling leaflets and electronic PAP. At the end of the study, the working groups were to organize a feedback discussion in the health centre, and they were encouraged to hold a final seminar locally.

### Evaluation

#### Process evaluation

The process evaluation examined the function of the four working groups in order to explain the accomplishment of the study goals. The evaluation focused on how the responsibilities of the development work were shared between the working group members and on the implementation of development actions.

*In evaluating development responsibilities*, the most important issues were the multi-professionalism of the working group, number of tutorials and work meetings held, participation of the working group members in tutorials and time allocated by the working groups for the development work. *In evaluating implementation of development actions*, the issues were the selection of patient group, internal and external (outside health sector) collaboration of the working group, contents of development actions, organizing feedback discussion at the end of the study and continuation of the development work. Data for process evaluation were obtained from the observation notes of the tutor and from the working groups’ meeting minutes on tutorials and work meetings.

#### Outcome evaluation

The outcome of the development work was evaluated with 19 variables (1–19), which reflected reaching of the five goals (i-v) of the study (Table 2). Outcome evaluation thus focused on the changes in PA counselling from the beginning of the 6-month development work (baseline) to the point it had ended (follow-up). The changes were examined from health professionals’, their patients’ and external partners’ viewpoint with the same measures at each health centre at baseline and follow-up. Table 2 introduces the measures, which were used in evaluating the changes in outcome variables in relation to each goal.

**Table 2:** Outcome evaluation of the study base on questionnaire to the health professionals (Additional file 1), record sheet on patient visits (Additional file 2), questionnaire to the patients (Additional file 3) and external partner interview (Additional file 4)

Goals, outcome variables and measures	Baseline %	Follow-up %	Change in %-points (95 % CI)
i) To increase know-how of health-related physical activity (PA) and PA counselling			
1. Proportion of professionals responding correctly to ten statements about health-enhancing PA.			
- questionnaire to the health professionals^a^	12	24	+12 (−1.6 to 25.6)
2. Proportion of professionals reporting that they have no deficiencies in the three items describing the know-how of PA recommendations and health benefits of PA.			
- questionnaire to the health professionals	27	44	+17 (−0.5 to 34.5)
3. Proportion of professionals reporting that they have no deficiencies in the four items describing the know-how of PA counselling			
- questionnaire to the health professionals	5	15	+10 (−0.6 to 20.6)
ii) To increase implementation and quality of PA counselling			
4. Proportion of professionals reporting that they give PA advice to at least two thirds of their patients.			
- questionnaire to the health professionals	40	38	−2 (−2.0 to −18.7)
5. Proportion of patient visits, where the professionals had given instructions on PA to the patient.			
- record sheet on patient visits^b^	44	38	−6 (−10.4 to −1.6)
6. Proportion of patients reporting that PA was discussed during the visit to the professional.			
- questionnaire to the patients^c^	54	54	0 (−7.1 to 7.1)
7. Proportion of respondents reporting that the four important PA issues were always discussed during the visit			
- questionnaire to the health professionals	1	4	+3 (−3.2 to 9.2)
- questionnaire to the patients	7	5	−2 (−5.6 to 1.6)
iii) To increase familiarity with and use of Physical Activity Prescription (PAP)			
8. Proportion of respondents reporting that they know what PAP is.			
- questionnaire to the health professionals	56	95	+39 (25.5 to 52.5)
- questionnaire to the patients	17	21	+4 (−1.9 to 9.9)
- external partner interview^d^	65	70	+5 (−19.5 to 29.5)
9. Proportion of professionals reporting that they have used PAP in their work.			
- questionnaire to the health professionals	5	37	+32 (18.9 to 45.1)
10. Proportion of professionals reporting that they have used PAP in their work during the past two weeks.			
- questionnaire to the health professionals	5	14	+9 (−1.5 to 19.5)
11. Proportion of visits where the professional had completed PAP.			
- record sheet on patient visits	0	4	+4 (2.7 to 5.3)
12. Proportion of patients reporting that PAP was completed during the visit.			
- questionnaire to the patients	4	5	+1 (−2.2 to 4.2)
13. Proportion of respondents reporting that they had an agreement on using PAP in their working unit.			
- questionnaire to the health professionals	0	32	+32 (20.3 to 43.7)
- external partner interview	2	18	+16 (−1.9 to 33.9)
iv) To increase internal and external collaboration in PA counselling			
14. Proportion of professionals reporting that they referred their patients for PA counselling to other health professionals sometimes or always.			
- questionnaire to the health professionals	83	80	−3 (−16.6 to 10.6)
15. Proportion of visits where the professional had used PAP as a referral to other health professional.			
- record sheet on patient visits	0	1	+1 (0.3 to 1.7)
16. Proportion of professionals reporting that they referred their patients sometimes or always to professionals outside health care in PA issues.			
- questionnaire to the health professionals	84	91	+7 (−4.8 to 18.8)
17. Proportion of external partners reporting that they collaborated with health centre in PA counselling.			
- external partner interview	67	75	+8 (−15.7 to 31.7)
v) To increase the use of electronic patient record systems in PA counselling			
18. Proportion of professionals reporting that they always entered information on PA discussions to the patient record system.			
- questionnaire to the health professionals	37	48	+11 (−5.8 to 27.8)
19. Proportion of visits where the professionals entered PA issues to the patient record system.			
- record sheet on patient visits	27	25	−2 (−6.0 to 2.0)

Study goals (i, ii, iii, iv, v), outcome variables (1–19) and measures (in italics). Proportions (%) of responses before (baseline) and after the development work (follow-up) and changes in percentage points (%-point) and their 95 % confidence intervals (95 % CI) from baseline to follow-up

^a^Number of respondents: Baseline 75, follow-up 80

^b^Number of patient visits: Baseline 1008, follow-up 1000

^c^Number of respondents: Baseline 441, follow-up 431

^d^Number of respondents: Baseline 48, follow-up 28

The research institute planned the evaluation procedure and measures and was responsible for analysing the data but the health centres carried out the practical arrangements. The measures used were a questionnaire to health professionals, a record sheet to health professionals on patient visits, a questionnaire to patients, and external partner interviews. All subjects were informed about the voluntary participation in the study.

The *questionnaire to health professionals* (Additional file 1) was addressed to all physicians, nurses and physiotherapists working in the health centres. The questionnaire to health professionals was the only measure, which was used in assessing the accomplishment of all goals and included outcome variables 1, 2, 3, 4, 7, 8, 9, 10, 13, 14, 16, 18. The responses given at baseline were outlined in the beginning of the study in the joint orientation meeting of the working group and steering group to help them to focus the development work. To achieve maximum response rate the questionnaire included no identification and was therefore delivered to all physicians, nurses and physiotherapists at both measurement points. As no identification was used some of the professionals may have responded only to baseline or follow-up questionnaire. Thus, the respondents at baseline and follow-up may have been slightly different.

Physicians, nurses and physiotherapists were also instructed to complete a *record sheet* (Additional file 2) after each non-emergency patient visit during five days or until they had recorded at least 15 patient visits. After each individual patient they were to answer ‘yes’ or ‘no’ to the following points: 1) Did the patient have a health problem that could be alleviated with PA? 2) Did you discuss PA with the patient? 3) Did you give instructions on PA to the patient? 4) Did you use PAP? 5) Did you use PAP as a referral? 6) Did you agree on follow-up visits with the patient?, 7) Did you enter information on PA to the patient record system? The health professionals completed the record sheets anonymously – only their professional group could be identified. Furthermore, the patients, whose visits the health professionals recorded, were presumably different at baseline and follow-up. Therefore, the change in PA counselling was not evaluated within the individual health professional or patient. The record sheet on patient visits evaluated goals ii), iii), iv) and v) and included outcome variables 5, 11, 15 and 19.

In addition to assessing the overall change in PA counselling among health professionals, the record sheets on patient visits were also used to evaluate change separately in each professional group (physicians, nurses, physiotherapists). This was because the record sheet data contained the largest number of observations by each professional group. The dataset was also considered more accurate than the questionnaire to health professionals because it had been accumulated after patient visits and might thus have contained less recollection bias. In the professional group analysis, the data were limited to visits where the professional had answered ‘yes’ to the question whether the patient had a health problem that could be alleviated with PA. This was because counselling was considered especially important in this subgroup of patient visits.

After the visit the health professionals were asked to give a *questionnaire to patients* (Additional file 3) to all patients they had included in the record sheet regardless of whether they had discussed PA or not with them to examine how PA counselling was implemented from the patient’s viewpoint. It is likely that most of the patients at the two measurement points (baseline and follow-up) were different. Therefore, the changes in PA counselling practices were not tracked within individual patients. The questionnaire to patients evaluated goals iii) and iv) and included outcome variables 6, 7, 8, 12.

PA counselling collaboration across sector boundaries was evaluated with *external partner interviews* (Additional file 4) carried out over the phone. They were targeted at local partners that the working group had named as the most potential stakeholders in PA counselling collaboration. These partners included, for example, municipal PA services, community centres, sports clubs and private fitness centres. All partners contacted at baseline were contacted again at follow-up. The external partner interviews evaluated goals iii) and iv) and included outcome variables 8, 13, 17.

### Statistical methods

The impact of the development work was analysed by comparing the proportions (%) of responses of each outcome variable at baseline and follow-up in a dataset including all four health centres. The difference in percentage points between baseline and follow-up represented change. To determine statistical significance of the changes, 95 % confidence intervals (CI) were calculated by using Wald confidence interval with continuity correction. The change was considered statistically significant (*p* < 0.05) if the 95 % CI values did not cross point zero. Also, the location of values in relation to zero was considered in interpreting the findings [28].

## Results

### Participants

Four health centres from the Pirkanmaa region, Finland participated in the study. They served 12000, 12000, 21000 and 22000 residents. The total number of health professionals belonging to the target group (physicians, nurses, physiotherapists) was 141 ranging from 29 to 47 per health centre. The description of datasets in relation to various measures (questionnaire to health professionals and patients, record sheets on patient visits, external partner interviews) at baseline and follow-up is provided in Table 3.

**Table 3:** Description of datasets in relation to each measure before (baseline) and after the development work (follow-up)^a^

	Baseline	Follow-up
Health professionals responding to the questionnaire, *N* (%) ^b^	75 (53 %)	80 (57 %)
Profession		
physician	18 (24)	13 (16)
nurse	42 (56)	43 (54)
physiotherapist	15 (20)	24 (30)
Years in the current working place, mean (SD)	11.3 (10.4)	11.2 (10.0)
Working in outpatient health services^c^	74 (99)	79 (99)
Patients per day, mean (SD)	9.0 (5.6)	12.7 (23.5)
Minutes per patient, mean (SD)	36.5 (18.3)	37.6 (18.7)
Patient visits on the record sheets, *N*	1008	1000
By a professional group, *N* (%)		
physician	413 (41)	228 (23)
nurse	417 (41)	633 (63)
physiotherapist	178 (18)	139 (14)
By a single employee, mean (SD)	9.1 (8.0)	9.1 (6.9)
physician	11.0 (9.5)	10.0 (8.1)
nurse	8.0 (6.6)	9.3 (6.5)
physiotherapist	7.0 (6.1)	7.2 (6.1)
Health professionals completing the record sheet on patients visits^d^	*N* = 112 (79 %)	*N* = 89 (63 %)
physician	35 (31)	17 (19)
nurse	51 (46)	54 (61)
physiotherapist	26 (23)	18 (20)
Patients responding to the questionnaire^e^	*N* = 441 (44 %)	*N* = 431 (43 %)
Age, mean (SD)	54.1 (17.8)	58.6 (17.3)
Sex		
female	286 (65)	280 (65)
male	153 (35)	148 (35)
Professional visited		
physician	193 (44)	198 (46)
nurse	152 (35)	135 (32)
physiotherapist	81 (19)	87 (20)
other	10 (2)	8 (2)
Reason for the visit		
diagnostic examinations or treatment of a symptom, disease or injury	261 (60)	277 (64)
preventive health examination	54 (12)	55 (13)
birth control, maternity care	35 (8)	27 (6)
other	85 (20)	71 (17)
First visit	152 (35)	128 (30)
Illness diagnosed by a physician^f^	338 (83)	350 (87)
Moderate-intensity aerobic physical activity 1–2 hours a week and muscular strength training on at least 2 days a week.	137 (33)	146 (37)
External partners interviewed	*N* = 48	*N* = 28
Primary working place		
non-profit health or patient organization	8 (17)	6 (21)
exercise and sports club	7 (15)	4 (14)
public PA services	7 (15)	2 (7)
private fitness centre	6 (12)	2 (7)
adult education centre etc.	4 (8)	3 (11)
other	16 (33)	11 (39)
Implements PA counselling in own work.	20 (42)	9 (32)
Reports that there is an agreement in the municipality about the coordination of health-enhancing PA.	24 (50)	18 (64)
Reports that there is an agreement in the municipality about sharing the responsibilities of health-enhancing PA.	15 (31)	8 (29)

Numbers (N) and proportions (%) or means and standard deviations (SD)

^a^In the datasets of questionnaire to health professionals and external partner interview the follow-up measures were addressed not only to those responding at baseline but also to non-respondents. As a result, the persons responding at baseline and follow-up may have been slightly different. In the datasets concerning record sheets on patient visits and questionnaire to the patients it is likely that majority of the patients were different at the two time points

^b^Questionnaire to the health professionals was targeted to physicians, nurses and physiotherapists, *n* = 141 at baseline and follow-up

^c^Multiple responses were allowed. If at least one of the responses was maternity or child health care, school or student health care, occupational health care, consultations for special groups, it indicated working in outpatient health services

^d^The record sheet on all non-emergency patient visits was delivered to physicians, nurses and physiotherapists (*n* = 141) to be kept for 5 days or until records on 15 patients had been completed

^e^The percentage has been calculated from the total number of patient visits recorded, which was 1008 at baseline and 1000 at follow-up

^f^Coronary artery disease, hypertension, claudication, other cardiovascular disease, high cholesterol, overweight, asthma, type 1 diabetes, type 2 diabetes, lower limb osteoarthritis, rheumatoid arthritis or other inflammatory joint disease, chronic or frequent low back pain, chronic or frequent neck-shoulder pain, osteoporosis or related fractures, low state of mood or depression, sleeping disorders, breast cancer, colon cancer

### Process evaluation

#### Development responsibilities

The four working groups involved 24 members altogether: 13 nurses, six physiotherapists and five physicians. Each working group, with number of members varying between 5 and 8, had one or several representatives from each professional group. The health centre management was represented regularly in only one and irregularly in one working group. Personnel from other settings were included in two working groups (persons from municipal PA services and community centre).

Altogether six to eight tutorial meetings were held for the working groups. Only eight (33 %) out of the 24 members of the working groups participated in all meetings. At each health centre altogether 12 to16 h were used for tutorials. In addition, the working groups at each health centre had 1 to 4 work meetings where issues agreed in tutorials were prepared. Usually only a few members of the team participated in these work meetings, which lasted from half an hour to two hours. None of the members had the chance to use 2 to 4 h per week on development work on a regular basis, as was suggested at the beginning of the study.

#### Implementation of development actions

Two health centres targeted the development of PA counselling at patients with Type 2 diabetes; one health centre at individuals arriving for 40-year preventive health examinations; and one health centre at mothers of small children. Internal collaboration in PA counselling increased in all health centres. In the health centre where the member of management was regularly present in the working group, the 3 commitment to the development work in terms of the participation of working group members was stronger than in the other health centres. In the two working groups, which included representatives from municipal PA services or community centre, collaboration between them and the health centre (external collaboration) increased leading to the initiation of a PA counselling service path: In one health centre regular PA counselling hours were organised by municipal PA services, and in the other health centre tailored group exercise was arranged for the mothers of small children by the community centre.

In addition to the 2-hour training session held by the researchers at the beginning of the study only one of the working groups arranged training on PA or PA counselling at their health centre during the development work. The training was on how to complete PAP and it was provided for the physicians during their weekly meeting. Three health centres succeeded in changing the way to enter information on PA counselling to the electronic patient record system by modifying the record template and user authorisations. In all health centres, the working groups were provided with supportive material for PA counselling, but its use was not explored.

All working groups implemented feedback discussions after the study. The number of persons in the discussions and their connection to the study varied between health centres: Some included only members of the working group and steering group, others involved also health centre personnel and stakeholders outside the study context. The development work continued in all health centres: in one, group exercise organised for the mothers of small children was continued with external funding; in another, the PA counselling model used in health examinations was extended to all scheduled appointments of the professionals; and in two health centres, the already implemented operations were continued.

### Outcome evaluation

Table 2 shows the outcome variables of the five study goals at baseline and follow-up, as well as their changes from baseline to follow-up.

i) To increase know-how of health-related PA and PA counselling (outcome variables 1–3)
  At baseline approximately a tenth (12 %) of the professionals gave a correct answer to ten of the 16 statements concerning health-related PA and a quarter (27 %) reported being familiar with the recommendations and health benefits of PA. Only 5 % of the professionals perceived deficiencies in their know-how related to PA counselling. At follow-up the know-how had increased 10–17 %-points depending on the outcome variable but none of the increases were statistically significant (Table 2).
ii) To increase implementation and quality of PA counselling (outcome variables 4–7)
  At baseline 40 % of the professionals reported that they gave PA advice to at least two thirds of their patients. The record sheets on patient visits indicated similarly that PA was discussed during 44 % of the visits. Every other patient (54 %) recalled that PA issues had been brought up at the visit. However, based on the questionnaire to patients only 1 % of the discussions included the four issues considered essential to PA counselling. The corresponding proportion in the questionnaire to professionals was 7 %. At follow-up the proportion of professionals giving PA advice and the proportion of visits including PA discussions decreased statistically significantly 2 and 6 %-points, respectively (Table 2). The other variables related to the implementation and quality of PA counselling did not change.
iii) To increase familiarity with and use of PAP (outcome variables 8–13)
  At baseline more than half (56 %) of the professionals, two thirds (65 %) of the external partners and a sixth of the patients (17 %) were familiar with PAP. However, only 5 % of the professionals had used it during the past two weeks or in general and it had not been used at any patient visit according to the record sheet. Even so, 4 % of the patients recalled receiving PAP during their visit to health professional. None of the health professionals and just 2 % of the external partners reported that they had a mutual agreement in their working unit to use PAP in PA counselling. At follow-up the familiarity of PAP among professionals had increased 39 %-points, usage 32 %-points and agreement on using it in PA counselling 32 %-points (Table 2). Patient visits with PAP were recorded 4 %-points more than at baseline. The results from the patient questionnaire and the external partner interviews were to the same direction but not statistically significant.
iv) To increase internal and external collaboration in PA counselling (outcome variables 14–17)
  At baseline majority of professionals reported referring their patients for PA advice to other health professionals in the centre (internal collaboration) or external partners (83 % and 84 %, respectively). Based on the record sheets on patient visits PAP was not used as a form of referral at any of the visits. Two-thirds (67 %) of the external partners reported collaborating with the local health centre in health-related PA issues. At follow-up the use of PAP as health professionals’ internal referral had increased statistically significantly 1 %-point (Table 2). No changes were discovered in other variables although the tendency seemed positive in professionals’ referrals to external partners and external partners’ collaboration with health centres.
v) To increase the use of electronic patient record systems in PA counselling (outcome variables 18–19)
  At baseline a little more than a third (37 %) of health professionals reported always entering information on PA to the patient record system. Based on the record sheets on patient visits, PA information was recorded to the system in a fourth (27 %) of the visits. At follow-up the proportion of professionals, who reported that they always recorded information on PA to the electronic system, had increased 11 %-points but the change was not statistically significant (Table 2).

#### PA counselling within professional groups

**Fig. 3:**
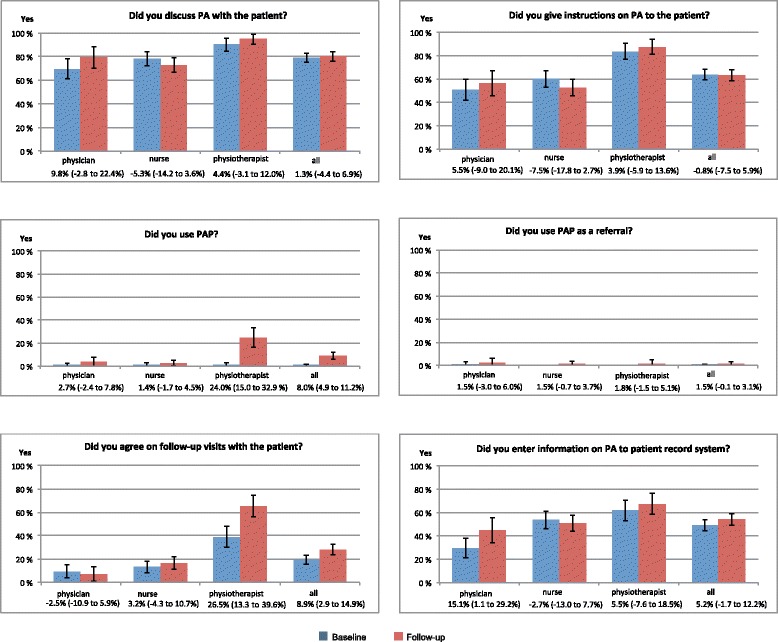
Physical activity (PA) counselling by the three professional groups (physician, nurse, physiotherapist) and in all professionals (all) at baseline and follow-up based on record sheet data on patient visits. The columns show the proportion (%) of professionals’ yes-answers to each particular question. The change from baseline to follow-up is presented under the name of each professional group and all professionals in percentage points and 95 % confidence intervals (in brackets). Data are limited to the visits where the professionals had evaluated that the patient had a health problem, which could be alleviated with PA. The number of this type of visits varied from 441 to 443 (44 %) at baseline and from 401 to 406 at follow-up (40 %) depending on the particular question

The total number of visits recorded to the record sheets was 1008 at baseline and 1000 at follow-up (Table 3). In 44 % of the baseline and 40 % of the follow-up visits the professionals had answered ‘yes’ to the question whether the patient had a health problem that could be alleviated with PA. In this subsample of visits among physiotherapists the use of PAP had increased 24 %-points, and arranging of follow-up visits 27 %-points from baseline to follow-up (Fig. 3). Among physicians, entering PA issues to the electronic patient record system had increased 15 %-points. No statistically significant changes were discovered among nurses.

## Discussion

### Summary of key findings

The development work succeeded the best in its third goal to increase the familiarity and use of PAP. This conclusion can be drawn from the fact that the directions of change were positive in all outcome variables related to the goal and also statistically significant in variables concerning the familiarity with PAP, use of PAP and agreement on using PAP. However, the changes from baseline to follow-up in the variables reflecting the use of PAP were smaller based on the record sheets on patient visits (+4 %-points) compared to those based on the questionnaire to the professionals (+32 %-points in the overall use and +9 %-points in the use during the past 2 weeks). This may indicate that some over-reporting happened with the questionnaire or that information was not completed in the record sheets after each patient who received PAP. A positive trend was also seen in the variables reflecting the know-how of health-related PA and PA counselling but none of the changes reached statistical significance.

In contrast, the results for other goals such as in increasing internal and external collaboration and use of electronic patient record systems in PA counselling were more modest. Even a change to an undesirable direction seemed to have taken place as the proportion of professionals always giving PA advice to their patients and the proportion of visits including PA discussion decreased from baseline. One possible explanation to this may be that after the training arranged to the health professionals in the beginning of the study and during the course of the development work the health professionals became more aware of what counselling actually involved and were therefore more critical about their responses concerning the frequency of counselling in the follow-up questionnaire. In relation to the quality of counselling it was alarming that only a small fraction of the professionals (7 %) and patients (1 %) reported at baseline that the four most important PA issues were always discussed during the visits. No change was observed at follow-up, which implicates that the quality issues need particular attention when developing PA counselling practices in the future.

According to the process evaluation the responsibilities for the development work were unevenly distributed between the members of the working groups: the working groups were multi-professional in principle but in practice only few active members were in charge of the concrete development work. Also the commitment of management was weak and collaboration with external partners limited as seen from their mild participation in the working groups. In addition, none of the working groups was able to regularly allocate the weekly minimum of two hours of working time for the development work, which may have been one reason for the scarce number of development actions taken. In the light of all this it seems surprising that the development work had continued after the study to some extent in all health centres.

### Comparison with findings from other studies

There is a lack of implementation research conducted in health care [29]. The majority of PA counselling studies have focused on examining the effects of PA counselling on patients’ PA behaviour (e.g. [30]) or they have studied the effects of counselling training on health professionals’ counselling practices (e.g. [31–33]). Less is known about how to transfer effective PA counselling approaches into health care routines [34].

A recent Australian study [35] aimed at increasing health professionals’ lifestyle counselling on smoking, eating fruit and vegetables, alcohol consumption and PA. Among other actions, the intervention included modifications to the patient record system, provision of new counselling materials, training for the staff, improvements to referral procedures, and monitoring of counselling with monthly reports. The persons in charge were trained and supported with meetings, phone contacts, emails, and resource websites. Phone interviews of patients showed that after the intervention the professionals more often asked about the patients’ PA habits and brought up the PA issues when compared to before the intervention, but the frequency of arranging follow-ups or referring patients to other professionals for additional guidance did not change. Thus, the results of the Australian study were quite similar to those in the present study.

### Strengths and limitations

The most important strengths of the study were i) the inclusion of a systematic process and outcome evaluation, ii) tailoring the development work according to the needs of individual health centre, and iii) the primary responsibility of the implementation being in the health centres themselves. These aspects are critical elements of implementation research [26] and may improve the applicability value of the results.

The strength of the present study was also the wide selection of measures in the outcome evaluation and that the outcome variable were multifaceted in nature, both enabling the examination of reaching the goals from different perspectives. The idea was that a change discovered with different measures in various outcome variables reflecting the same goal would strengthen the evidence on reaching the particular goal. Multisided evaluation was particularly important because there was no previous information about the reliability of the measures used in assessing change.

Using several measures does not, however, rule out reliability issues related to single measures. It is known, for instance that self-reports are susceptible to overestimations among health professionals [36]. This may result from e.g. recollection bias and desire for social acceptability [37]. The questionnaire to the professionals was therefore complemented with record sheets on patient visits and a questionnaire to the patients, which were both instructed to be completed after the appointments. However, the questionnaire data from the patients visiting a health professional was obtained only from 44 % of the patients, which may refer to some kind of selection bias and weaken the reliability of the findings. It may be that the patients were too busy to fill out questionnaires after the appointment. Nevertheless, audit charts and patient surveys have been shown to have better validity than self-reports in physicians [36] and they are moderately valid when compared to direct observation, which is considered the golden standard in counselling studies [38]. Applying direct observation or audio taping would undeniably have improved the validity of evaluation in regard to most outcome variables but unfortunately the study had no resources for this. Obtaining information from medical records was, on the other hand, not possible due to strict data protection related to patient record systems. The information on PA counselling could not have been extracted without reading patients’ entire medical information because it had no specific place in the record.

The interpretation of the results was hindered the most by the different sizes of the datasets collected with different measures. By viewing both baseline and follow-up measurements it can be seen that the sizes of the datasets varied from 28 to 48 in external partner interviews and from 1000 to 1008 in record sheets on patient visits. Therefore, a percentage difference that appeared large in the outcome variable of a small dataset did not reach statistical significance, while even a small difference in the outcome variable describing the same thing in a larger dataset was statistically significant. In addition, not using identification in health professionals’ questionnaire prevented from evaluating the changes from baseline to follow-up within the same group of health professionals. On the other hand, the training and development work was supposed to target the whole staff and thus the changes in PA counselling, if there were any, should have been observed at follow-up regardless of the respondents. Identification may have caused suspicion and weakened the response rates. In the patient questionnaire or record sheet for patient visits, identification of the patients was not meaningful since it was anyway likely that majority of the patients visiting the health professionals at baseline and follow-up were different. The purpose was to see the general changes in PA counselling from the patients’ viewpoint, not the practices related to particular patients. The poorer response rate especially among the physicians at follow-up compared to the baseline is also a concern, which may have affected the validity of the results.

The outcome evaluation was also made difficult by the different sizes of the health centres, as data collected from larger health centres may have received more weight in the analysis. This could weaken the generalizability of the results. The outcome evaluation may well be criticized for the fact that it was not conducted in those patient groups, which the health centres had chosen as targets for their development work. It may be that by analysing the changes in the selected target group the results may have appeared more positive. The separate analysis on record sheet data, which shows the change in PA counselling within each health professional group (Fig. 3), may better ‘match’ to the patient groups, which were the targets of the working groups’ development work. This is because it included only those visits where the professional had answered ‘yes’ to the question whether the patient had a health problem that could be alleviated with PA.

In addition, the study lacked a comparison group, which prevents from making direct conclusions that the findings resulted from the development work. However, forming a nonrandomized comparison group from a few additional health centres would not have improved the setup. And even if there have been more centres, matching them to an intervention and a comparison group would have been problematic due to their diversity. In this respect pragmatic proposals such as made by Leykum et al. [26] about how participatory action research can be integrated into randomized controlled trials and by Glasgow et al. [39] about how research can be translated to the practice through more pragmatic study designs are needed especially in implementation studies involving healthcare organizations.

Finally, the timeframe for the development work was only 6 months. It is likely that longer period of time would have been needed especially to achieve changes at organizational level [40].

## Conclusions

This study is an example of implementation research, which aimed at developing PA counselling practices in primary care’s everyday routines. It also shows one approach to evaluate the process and outcomes of the implementation.

The development work of the working groups was able to achieve some changes in the familiarity with and use of PAP, and with less extent in the know-how of health-related PA and PA counselling. Changes in other goal-specific outcome variables, which are more related to organisational issues (e.g. time allocation, management commitment), inter-professional agreements (e.g. entering information to patient record system, referral practice) and systematic inter-sectorial collaboration (e.g. with municipal PA services), may have required longer timeframe. Also more actions may have been needed to remove the most important obstacles for PA counselling. Generally they relate to the valuation of counselling, health professionals’ know-how on behaviour change counselling and time allocated for counselling [8, 12]. In this respect it may have been productive in this study and suggestible in similar studies in the future to examine the multilevel barriers of PA counselling and to utilize the findings in the development work.

To document and tackle the obstacles behind lifestyle counselling, more research is needed on the determinants [23, 27, 41], organizational factors [40, 42, 43] and collaboration [44] operationalizing the implementation of health promotion and more specifically of PA counselling in primary care.

## Additional files

Additional file 1: Outcome variables (1,2,3,4,7,8,9,10,13,14,16,18) as well as the questions (in italics) and their response alternatives (right column), which were used in evaluating the accomplishment of the goals of the study (i-v) on the basis of questionnaire to health professionals. The bolded response alternatives indicated accomplishment. (DOCX 24 kb)
Additional file 2: Record sheet on patient visits. (DOCX 102 kb)
Additional file 3: Outcome variables (6,7,8,12) as well as the questions (in italics) and their response alternatives (right column), which were used in evaluating the accomplishment of the goals of the study (ii and iii) on the basis of questionnaire to the patients. The bolded response alternatives indicated accomplishment. (DOCX 19 kb)
Additional file 4: Outcome variables (8,13,17) as well as the questions (in italics) and their response alternatives (right column), which were used in evaluating the accomplishment of the goals of the study (iii, iv) on the basis of external partner interview. The bolded response alternatives indicated accomplishment. (DOCX 18 kb)

## Abbreviations

PA: Physical activity
PAP: Physical Activity Prescription
PAPP: National Physical Activity Prescription Program
