# Supplementary material for: Developing physical activity counselling in primary care through participatory action approach
Source: BMC Fam Pract. 2016 Oct 4;17:141. doi: 10.1186/s12875-016-0540-x (PMC5051097; doi:10.1186/s12875-016-0540-x)
Supplement: Additional file 1: — Outcome variables (1,2,3,4,7,8,9,10,13,14,16,18) as well as the questions (in italics) and their response alternatives (right column), which were used in evaluating the accomplishment of the goals of the study (i-v) on the basis of questionnaire to health professionals. The bolded response alternatives indicated accomplishment. (DOCX 24 kb) [file 12875_2016_540_MOESM1_ESM.docx]

Appendix 1. Outcome variables (1,2,3,4,7,8,9,10,13,14,16,18) as well as the questions (in italics) and their response alternatives (right column), which were used in evaluating the accomplishment of the goals of the study (i-v) on the basis of questionnaire to health professionals. The bolded response alternatives indicated accomplishment.

| **Study goals, outcome variables and questions** | **Response alternatives** |
| --- | --- |
| **i) To increase know-how of health-related physical activity (PA) and PA counselling** |  |
| 1. Proportion of professionals responding correctly to ten statements about health-enhancing PA.  *In your opinion, are the following statements true or false? To achieve health benefits one PA session has to last at least one hour (F); PA recommendations includes also muscular training at least twice a week (T); To achieve health benefits PA should cause severe breathlessness (F); Regular PA is not necessary because the benefits of PA sustain for a long time (F); PA can not be considered medicine (F); Some PA is better than being totally physically passive (T); PA should be reduced during pregnancy (F); Medical check-up should always be performed before starting to exercise (F); PA benefits health even without losing weight (T); Two and half hours of brisk walk in a week is sufficient for health (F).* | 1= True (T)  2= False (F)  3= No comment |
| 2. Proportion of professionals reporting that they have no deficiencies in the three items describing the know-how of PA recommendations and health benefits of PA.  *How do you rate your know-how in PA counselling? Take stand to the following statements: I know PA recommendations; I know the health benefits of aerobic PA; I know the health benefits of muscular training.* | **1= I have no deficiencies**  2= I have some deficiencies  3= I have a lot of deficiencies |
| 3. Proportion of professionals reporting that they have no deficiencies in the four items describing the know-how of PA counselling.  *How do you rate your know-how in PA counselling? Take stand to the following statements: I am able to give information on health risks of inactivity; I am able to enhance behaviour change; I am able to support compliance with follow-up; I am able to give advice on suitable quality and quantity of PA.* | **1= I have no deficiencies**  2= I have some deficiencies  3= I have a lot of deficiencies |
| **ii) To increase implementation and quality of PA counselling** |  |
| 4. Proportion of professionals reporting that they give PA advice to at least two thirds of their patients.  *To how many patients do you give advice on PA?* | **❒ To all**  **❒ To approx. two thirds**  ❒ To approx. every other one  ❒ To every third  ❒ To less than third or no-one |
| 7. Proportion of respondents reporting that the four important PA issues were always discussed during the visit.  *How often do you discuss following PA issues with your patients? Current PA habits; Possibility to be physically active in terms of life situation and environment; Patient’s own PA goals; Need for follow-up.* | 1= Never  2= Sometimes  **3= Always** |
| **iii) To increase familiarity with and use of Physical Activity Prescription (PAP)** |  |
| 8. Proportion of respondents reporting that they know what PAP is  *Do you know what PAP is?* | ❑ No  ❑ I have heard the name but I do not exactly know what it is.  **❑ Yes, I know what PAP is but I have not used it myself.**  **❑ Yes, I know what PAP is and I have used it in my work.** |
| 9. Proportion of professionals reporting that they have used PAP in their work.  *Do you know what PAP is?* | ❑ No  ❑ I have heard the name but I do not exactly know what it is.  ❑ Yes, I know what PAP is but I have not used it myself.  **❑ Yes, I know what PAP is and I have used it in my work.** |
| 10. Proportion of professionals reporting that they have used PAP in their work during the past two weeks.  *Have you used PAP in your work during the past two weeks?* | ❑ No  **❑ Yes** |
| 13. Proportion of respondents reporting that they had an agreement on using PAP in their working unit.  *Is PAP used in your working unit?* | ❑ No  ❑ I do not know.  ❑ Yes, irregularly or by only few persons.  **❑ Yes, we have agreed on using PAP.** |
| **iv) To increase internal and external collaboration in PA counselling** |  |
| 14. Proportion of professionals reporting that they referred their patients for PA counselling to other health professionals sometimes or always.  *Do you refer your patients to other health professionals in PA issues?* | 1= Never  **2= Sometimes**  **3= Always** |
| 16. Proportion of professionals reporting that they referred their patients sometimes or always to professionals outside health care in PA issues.  *Do you refer your patients to outside health centre (e.g. PA services, adult education centre, sports club, private fitness centre)?* | 1= Never  **2= Sometimes**  **3= Always** |
| **v) To increase the use of electronic patient record systems in PA counselling** |  |
| 18. Proportion of professionals reporting that they always entered information on PA discussions to the patient record system.  *Do you enter information on PA discussions to the patient record system?* | 1= Never  2= Sometimes  **3= Always** |
