# Supplementary material for: Developing physical activity counselling in primary care through participatory action approach
Source: BMC Fam Pract. 2016 Oct 4;17:141. doi: 10.1186/s12875-016-0540-x (PMC5051097; doi:10.1186/s12875-016-0540-x)
Supplement: Additional file 2: — Record sheet on patient visits. (DOCX 102 kb) [file 12875_2016_540_MOESM2_ESM.docx]

Appendix 2. Record sheet on patient visits.

| Patient | Did the patient have a health problem that could be alleviated with PA? | | Did you discuss PA with the patient? | | Did you give instructions on PA to the patient? | | Did you use PAP? | | Did you use PAP as a referral? | | Did you agree on follow-up visits with the patient? | | Did you enter information on PA to the patient record system? |
| --- | --- | --- | --- | --- | --- | --- | --- | --- | --- | --- | --- | --- | --- |
| 1 | ❒ yes ❒ no | | ❒ yes ❒ no | | ❒ yes ❒ no | | ❒ yes ❒ no | | ❒ yes ❒ no | | ❒ yes ❒ no | | ❒ yes ❒ no |
| 2 | ❒ yes ❒ no | | ❒ yes ❒ no | | ❒ yes ❒ no | | ❒ yes ❒ no | | ❒ yes ❒ no | | ❒ yes ❒ no | | ❒ yes ❒ no |
| 3 | ❒ yes ❒ no | | ❒ yes ❒ no | | ❒ yes ❒ no | | ❒ yes ❒ no | | ❒ yes ❒ no | | ❒ yes ❒ no | | ❒ yes ❒ no |
| 4 | ❒ yes ❒ no | | ❒ yes ❒ no | | ❒ yes ❒ no | | ❒ yes ❒ no | | ❒ yes ❒ no | | ❒ yes ❒ no | | ❒ yes ❒ no |
| 5 | ❒ yes ❒ no | | ❒ yes ❒ no | | ❒ yes ❒ no | | ❒ yes ❒ no | | ❒ yes ❒ no | | ❒ yes ❒ no | | ❒ yes ❒ no |
| 6 | ❒ yes ❒ no | | ❒ yes ❒ no | | ❒ yes ❒ no | | ❒ yes ❒ no | | ❒ yes ❒ no | | ❒ yes ❒ no | | ❒ yes ❒ no |
| 7 | ❒ yes ❒ no | | ❒ yes ❒ no | | ❒ yes ❒ no | | ❒ yes ❒ no | | ❒ yes ❒ no | | ❒ yes ❒ no | | ❒ yes ❒ no |
| 8 | ❒ yes ❒ no | | ❒ yes ❒ no | | ❒ yes ❒ no | | ❒ yes ❒ no | | ❒ yes ❒ no | | ❒ yes ❒ no | | ❒ yes ❒ no |
| 9 | ❒ yes ❒ no | | ❒ yes ❒ no | | ❒ yes ❒ no | | ❒ yes ❒ no | | ❒ yes ❒ no | | ❒ yes ❒ no | | ❒ yes ❒ no |
| 10 | ❒ yes ❒ no | | ❒ yes ❒ no | | ❒ yes ❒ no | | ❒ yes ❒ no | | ❒ yes ❒ no | | ❒ yes ❒ no | | ❒ yes ❒ no |
| 11 | ❒ yes ❒ no | | ❒ yes ❒ no | | ❒ yes ❒ no | | ❒ yes ❒ no | | ❒ yes ❒ no | | ❒ yes ❒ no | | ❒ yes ❒ no |
| Continuing… | | | | | | | | | | | | | |
| Remarks or comments: | | | | | | | | | | | |  |  |
|  |  |  | |  | |  | |  | |  | |  |  |
