# Supplementary material for: Developing physical activity counselling in primary care through participatory action approach
Source: BMC Fam Pract. 2016 Oct 4;17:141. doi: 10.1186/s12875-016-0540-x (PMC5051097; doi:10.1186/s12875-016-0540-x)
Supplement: Additional file 3: — Outcome variables (6,7,8,12) as well as the questions (in italics) and their response alternatives (right column), which were used in evaluating the accomplishment of the goals of the study (ii and iii) on the basis of questionnaire to the patients. The bolded response alternatives indicated accomplishment. (DOCX 19 kb) [file 12875_2016_540_MOESM3_ESM.docx]

Appendix 3. Outcome variables (6,7,8,12) as well as the questions (in italics) and their response alternatives (right column), which were used in evaluating the accomplishment of the goals of the study (ii and iii) on the basis of questionnaire to the patients. The bolded response alternatives indicated accomplishment.

| **Study goals, outcome variables and questions** | **Response alternatives** |
| --- | --- |
| **ii) To increase implementation and quality of PA counselling** |  |
| 6. Proportion of patients reporting that their current PA habits had been discussed during their previous visit to the health professional.  *Did you discuss your current PA habits during your previous visit to the health professional?* | ❒ No  **❒ Yes** |
| 7. Proportion of respondents reporting that the four important PA issues were discussed during their previous visit to health professional.  *Did you discuss about the following issues during your previous visit to health professional: Current PA habits; Possibility to be physically active in terms of life situation and environment; PA goals; Need for follow-up.* | ❑ No  **❑ Yes**  ❑ I do not remember |
| **iii) To increase familiarity with and use of Physical Activity Prescription (PAP)** |  |
| 8. Proportion of respondents reporting that they know what PAP is.  *Do you know what PAP is?* | ❒ No  ❒ Yes, I have heard the word but I do not exactly know what it is.  **❒ Yes, I know what PAP is but I have not received one myself.**  **❒ Yes, I know what PAP is and I have received one myself.** |
| 12. Proportion of patients reporting that PAP was completed during their previous visit to health professional.  *Did you complete PAP with the health professional during your previous visit?* | ❒ No  **❒ Yes** |
