# Supplementary material for: Developing physical activity counselling in primary care through participatory action approach
Source: BMC Fam Pract. 2016 Oct 4;17:141. doi: 10.1186/s12875-016-0540-x (PMC5051097; doi:10.1186/s12875-016-0540-x)
Supplement: Additional file 4: — Outcome variables (8,13,17) as well as the questions (in italics) and their response alternatives (right column), which were used in evaluating the accomplishment of the goals of the study (iii, iv) on the basis of external partner interview. The bolded response alternatives indicated accomplishment. (DOCX 18 kb) [file 12875_2016_540_MOESM4_ESM.docx]

Appendix 4. Outcome variables (8,13,17) as well as the questions (in italics) and their response alternatives (right column), which were used in evaluating the accomplishment of the goals of the study (iii, iv) on the basis of external partner interview. The bolded response alternatives indicated accomplishment.

| **Study goals, outcome variables and questions** | **Response alternatives** |
| --- | --- |
| **iii) To increase familiarity with and use of Physical Activity Prescription (PAP)** |  |
| 8. Proportion of respondents reporting that they know what PAP is.  *Do you know what PAP is?* | ❒ No  ❒ Yes, I have heard the word but I do not exactly know what it is.  **❒ Yes, I know what PAP is but I have not used it myself.**  **❒ Yes, I know what PAP is and I have used it in my work.** |
| 13. Proportion of respondents reporting that they had an agreement on using PAP in their working unit.  *Is PAP used in your working unit?* | ❑ No  ❑ I do not know.  ❑ Yes, irregularly or by only few persons.  **❑ Yes, we have agreed on using PAP.** |
| **iv) To increase internal and external collaboration in PA counselling** |  |
| 17. Proportion of external partners reporting that they collaborated with health centre in PA counselling.  *Does your working unit collaborate with health centre in PA counselling?* | ❑ No  **❑ Yes**  ❑ I can not say. |
